# Body Image and Body Mass Index Influence on Psychophysical Well-Being in Bariatric Patients: A Cross-Sectional Study

**DOI:** 10.3390/jpm12101597

**Published:** 2022-09-28

**Authors:** Virginia Campedelli, Chiara Ciacchella, Giorgio Veneziani, Irene Meniconzi, Emanuela Paone, Gianfranco Silecchia, Carlo Lai

**Affiliations:** 1 Department of Dynamic and Clinical Psychology and Health Studies, Faculty of Medicine and Psychology, Sapienza University of Rome, Via degli Apuli 1, 00185 Rome, Italy; 2 Department of Medical Surgical Sciences and Biotechnologies, Faculty of Pharmacy and Medicine, Polo Pontino, Bariatric Center of Excellence IFSO-EU, Sapienza University of Rome, Corso della Repubblica, 79, 04100 Latina, Italy; 3 Department of Medical and Surgical Sciences and Translational Medicine, Faculty of Medicine and Psychology, Sapienza University of Rome, Via di Grottarossa 1035, 00189 Rome, Italy

**Keywords:** body image, body mass index, weight, psychophysical well-being, bariatric surgery, obesity

## Abstract

Background: Psychophysical factors may have an impact on the disease of obesity, and it is important to explore which aspects may play an important role on the well-being of obese patients undergoing bariatric surgery. The purpose of this study was to assess the associations of a high body mass index (BMI) and greater dissatisfaction with body image with higher levels of psychopathological aspects, feelings of hopelessness, and psychological and physical health in patients undergoing evaluation for bariatric surgery. Methods: Fifty-nine patients undergoing bariatric surgery filled out the Symptom Checklist-90-Revised, the Body Uneasiness Test, the 12-item Short Form Survey, the Beck Inventory Scale II, and the Beck Hopelessness Scale. Correlations and hierarchical regressions between measures were performed. Results: Dissatisfaction with the perception of one’s own body image was strongly correlated with a worse psychophysiological health. On the contrary, BMI showed no significant correlation with the previous variables. Furthermore, the perception of one’s own body image significantly predicted the state of psychological health. Conclusions: The findings showed a more relevant role of body image compared to the BMI in the association with psychological outcomes, suggesting the importance of considering body image in the assessment and treatment of obese patients requiring bariatric treatment.

## 1. Introduction

Bariatric surgery is currently the gold standard for the treatment of morbid obesity and its comorbidities, including type 2 diabetes mellitus, arterial hypertension, dyslipidemia, cardiovascular disease, sleep-apnea–hypopnea syndrome, and cancer, leading to an improvement in psychophysical well-being [1]. This treatment should be considered for people with BMI ≥ 40 kg/m^2^ and for people with BMI ≥ 35–40 kg/m^2^ with associated comorbidities that are expected to improve with weight loss [2].

Obesity can significantly affect an individual and it is expected to have a negative clinical impact, influencing not only physical health, but also their psychological health, behavior, lifestyle, and overall quality of life [3,4]. These can be some of the main motivations for weight loss in individuals with obesity and some of the factors that motivate the decision for surgical treatment-seeking [5]. In fact, people with obesity believe that weight loss after bariatric surgery could improve their psychosocial well-being, allowing them the ability to live a normal life and to feel in control of their total health [5].

Psychophysical factors can impact obesity disease directly and indirectly [6], and it is important to explore which aspects may play an important role in the psychological and physical well-being of obese patients undergoing bariatric surgery.

Previous studies have investigated the association between body weight and psychological aspects in bariatric patients, showing that the improvement of these aspects following bariatric surgery appears to be directly linked to changes in body weight [7]. These results are supported by the fact that, after bariatric surgery, most patients report a greater improvement in psychosocial functioning and quality of life in concurrence with body weight loss [8]. Montpellier and colleagues [9] showed that patients undergoing Roux-en-Y Gastric Bypass surgery with a higher percentage of total weight loss showed an improvement in physical and psychosocial aspects at 24 months after bariatric surgery.

On contrary, other studies have shown that, despite weight loss, there is no improvement in psychological aspects in the long run. In fact, in a recent review, it was suggested that although bariatric surgery brought significant weight loss and improvement in medical co-morbidities, there was no evidence of improvement in the psychosocial aspects of patients in the long run, compared to nonsurgical management of obesity [8].

Spirou and colleagues [7] had to systematically review depression and anxiety outcomes at different time points following bariatric surgery and identify whether bariatric surgery significantly reduces psychological symptoms over time. The results suggest that depression is reduced for most participants in the first 24 months after bariatric surgery, while after 36 months of follow-up, depressive symptoms increase and may even return to pre-surgery levels [7]. The same results were found for anxiety: indeed, most patients undergoing bariatric surgery report a short-term reduction in anxiety, but over time, anxiety may increase and return always to pre-surgery levels [7].

This suggests that there may be factors underlying actual body weight that may influence the psychophysical well-being of people with obesity undergoing bariatric surgery.

Among the various factors involved, the perception of one’s body image may be one of the aspects that influence the mental health of people with obesity. Body image is a complex, multi-dimensional construct comprised of thoughts, emotions, perceptions, and evaluations relative to one’s own body [10], and concerns with one’s body image, including dissatisfaction with this, encompass shape and weight, including a fear of weight gain or figure due to excess skin [11].

The recent literature suggests how concerns about body image can significantly affect a person with obesity and focuses on the perception of one’s own body image after bariatric surgery, reporting different results. In general, obese patients pre- or post-bariatric surgery showed a clear association between body image perception and psychological state [12,13,14,15]. One recent study examined psychological well-being and body image in a sample of pre-bariatric surgery patients and in those with 1-year follow-up, showing a body image and psychological well-being improved in most patients [16].

Instead, a recent review on the goal of examining body image changes in patients with obesity pre- and post-bariatric surgery showed that, in some cases, the perception of one’s own body image does not change between pre- and post-bariatric surgery, suggesting that body image is not directly associated with the elevated BMI in obese patients [17]. This could be due to a difficulty to adapt one’s body image to the physical change due to the surgery. Other studies reveal how obese patients can suffer from psychiatric diseases such as depression and anxiety due to a severe dissatisfaction with their body image, even after bariatric surgery [14,15].

Furthermore, previous studies have shown how concern about one’s body image can lead, in conjunction with depressive aspects, to feelings of hopelessness that can further worsen the state of psychophysical well-being of obesity people [18,19].

However, the reasons for maintaining an obese identity and body image dissatisfaction despite weight loss are still unclear and existing studies in the literature show the complexity of this issue.

Given the discordance of the different studies, it is important to examine the role of the perception of one’s body image and body weight in relation to physical and psychological health as this could be important to understand the impact of psychological factors on weight loss, weight regain, and weight maintenance.

The aim of this study is to investigate which aspect between the perception of one’s own body image and body mass weight with body mass index can play an important role in relation to aspects of psychophysical health in a preliminary phase of bariatric surgery. In particular, the hypothesis of this study is that the dissatisfaction with their body image in obese people who undergo bariatric surgery can influence more negatively their psychophysical health, than their actual high body mass index.

## 2. Materials and Methods

### 2.1. Procedures

This study was conducted at the Bariatric Centre of Excellence IFSO-EC of “Sapienza” University of Rome, in collaboration with the Department of Dynamic and Clinical Psychology, and Health Studies of “Sapienza” University of Rome. The participants were recruited among patients attending the psychological assessment for bariatric surgery eligibility in the Bariatric Centre of Excellence IFSO-EC of “Sapienza” University of Roma, between November 2020 and July 2021. Informed consent was obtained from all individual participants included in the study. The recruitment was carried out by two trained clinical psychologists one month before surgery and the patients completed the self-reported anonymous questionnaires alone in a private room.

The inclusion criteria for participation in the study were to (a) be over 18 years of age; (b) have Italian nationality; (c) be eligible for surgery according to the European Guidelines on Metabolic and Bariatric Surgery for which bariatric surgery should be considered for patients with BMI ≥ 40 kg/m^2^, for patients with BMI ≥ 35–40 kg/m^2^ with associated comorbidities that are expected to improve with weight loss, and for patients with ≥ BMI 30–35 kg/m^2^ and type 2 diabetes and/or arterial hypertension with poor control despite optimal medical therapy [2]. The exclusion criteria were (a) presence of psychopathologies already diagnosed and under psychopharmacological treatment; (b) presence of drugs or alcohol abuse self-admitted; (c) undergoing primary bariatric procedures. The inclusion and exclusion criteria were evaluated during the preoperative psychological assessment.

This study was performed in line with the principles of the Declaration of Helsinki. Approval was granted by the Ethics Committee of the University Department of Dynamic and Clinical Psychology, and Health Studies of “Sapienza” University of Rome (11/27/20, No 0001118).

### 2.2. Measures

All the subjects underwent a psychological interview conducted by the clinical research psychologist to assess the presence/absence of inclusion and exclusion criteria; in addition, they were asked sociodemographic information such as gender, age, weight, height and BMI, marital status, education level, and work status. Anthropometric measurements were made using standard calibrated instruments: the height evaluated in meters was determined using a wall-mounted stadiometer and the weight evaluated in kilograms was determined by electronic scales. In contrast, BMI was calculated as weight in kilograms divided by the square of height in meters.

Subsequently, subjects who were eligible to participate in the study completed the following questionnaires. The Symptom Checklist-90-Revised (SCL-90-R) [20] is a widely used checklist that measures levels of psychological symptoms experienced in the past week, on a five-point Likert scale ranging from “not at all” to “extremely.” The SCL-90-R includes three global indexes: Global Severity Index (GSI) (designed to measure overall psychological distress), Positive Symptom Total (PST) (reports number of self-reported symptoms), and Positive Symptom Distress Index (PSDI) (designed to measure the intensity of symptoms). There are also nine symptomatologic sub-scales exploring the condition during the previous seven days: somatization, obsessive-compulsive, interpersonal sensitivity, depression, anxiety, hostility, phobic anxiety, paranoid ideation, and psychoticism. The Italian version was used [21] and the reliability coefficients of internal consistency were good for this study (Cronbach’s alpha values = 0.76/0.87). For the purpose of the present study, only the SCL-90-R GSI and the SCL-90-R PST were used.

The Body Uneasiness Test (BUT) [22] is a multidimensional tool for the clinical assessment of body image disorders and related psychopathologies. BUT is a 71-item self-report questionnaire that consists of two parts. BUT-A is a 34-item scale that indicates the presence of body uneasiness and investigates 5 scales: weight phobia (fear of being or becoming fat), body image concerns (overconcern with physical appearance), avoidance (body-image-related avoidance behaviors), compulsive self-monitoring (rituals involving checking physical appearance), and depersonalization (feelings of detachment or estrangement from one’s body). The whole score mean constitutes the Global Severity Index (GSI) that identifies the degree of severity related to body image. The severity rating is expressed on a scale from 0 to 5, where 0 corresponds to no problems in that area and 5 to maximum severity. The higher the indexes of each group, the greater the body uneasiness. Instead, BUT-B is a 37-item scale assessing specific worries about particular body parts, shapes, or functions. There are two evaluations resulting from this second part of the questionnaire: the overall result of the number of body parts for which one has worries with Positive Symptom Total (PST), and the index of overall discomfort with Positive Symptom Distress Index (PSDI). The severity rating is expressed in this case on a scale from 0 to 37, where 0 corresponds to the absence of the symptom and 37 corresponds to maximal body discomfort. The validity of BUT demonstrated good internal consistency (BUT-A Cronbach’s α values = 0.57–0.88; BUT-B Cronbach’s α values = 0.64–0.88).

The 12-item Short Form Health Survey (SF-12) [23] is a generic quality of life (QoL) instrument that includes a subset of 12 items from the 36-item Short Form Survey. The global measure is Total Score (SF-12 Total), with the subscales of Physical Health and Mental Health. Higher scores indicate an improvement in QoL. For this study, the Italian version was used [24] and the internal consistency reliability coefficients were good (Cronbach’s α value = 0.63).

The Beck Inventory Scale II (BDI II) [25] assesses the presence and severity of depressive symptoms. A total score above 10 is the beginning of the presence of depression. High scores show greater severity of depression. For this study, the Italian version was used [26] and it showed good internal consistency with a Cronbach coefficient α of 0.84.

The Beck Hopelessness Scale (BHS) [27] assesses the presence of hopelessness and negative expectations about the future, indicating the risk of suicide. It consists of 20 items in the form of yes-no questions and higher scores indicate more severe despair. For this study, the Italian version was used [28] and the internal consistency was good with a Cronbach’s α of 0.73.

### 2.3. Statistical Analyses

Continuous variables (weight and BMI) are presented as mean  ±  standard deviation, counts, or percentages. Categoric variables (education level, work, and marital status) are presented as counts and percentages.

Correlational analyses (Pearson’s r) between weight, BMI, sociodemographic characteristics, and the psychological outcomes (BUT, SCL-90-R GSI, SCL-90-R PST, SF-12 Physic Health, SF-12 Mental Health, BDI II, and BHS) were performed. Moreover, six hierarchical multiple-regression analyses with two steps were conducted to investigate the effects of BMI, BUT-A GSI, and BUT-B PSDI on psychological outcomes (SCL-90-R GSI, SCL-90-R PST, SF-12 Physic Health, SF-12 Mental Health, BDI II, and BHS). In hierarchical regression analysis, BMI was entered in step 1. BUT-A GSI and BUT-B PSDI were added in step 2. Only when significant correlations were found between sociodemographic characteristics and the psychological outcomes, a third step was planned, entering the sociodemographic characteristics in step 3.

The indicators provided in the regression models included R, R2, adjusted R2, F-value, Cohen’s f2 effect-size, R2 change, B, and standardization regression coefficient (β).

All analyses were performed with STATISTICA 8.0 software (StatSoft Inc., Tulsa, OK, USA) and the criterion for statistical significance was *p*  <  0.05.

A priori power analysis was conducted using G*Power 3.1 software (Düsseldorf, Germany) [29] considering the coefficient of determination (ρ2 = 0.27 with an α error probability of 0.001) reported by a previous study [30], which investigated the association between body image concerns and disability assessment in bariatric patients. The power analysis with the power set at 80% [31] indicated a required total sample size of 50 participants. Moreover, a post hoc power analysis was performed to compute expected achieved power using G*Power 3.1 software. Post hoc power analyses conducted considering the total sample size (*n* = 59), the minimum and maximum R2 levels (min R2 = 0.11 with F2 = 0.12; max R2 = 0.53, F2 = 1.31), an α error probability of 0.05, and three tested predictors resulted in a statistical power range from 0.56 to 1.00.

## 3. Results

A total of 59 participants were enrolled between November 2020 and July 2021. They were predominately female (*n* = 44; 74.6%) and the average age was 39.17 years (SD  =  10.11; range 19–55). Table 1 shows the participants’ sociodemographic and psychological characteristics.

The correlational analyses (Pearson’s r) among the weight, BMI, sociodemographic characteristics, and the psychological outcomes (BUT, SCL-90-R, SF-12, BDI II, and BHS) are reported in Table 2.

Gender was negatively correlated with weight, BMI, and SF-12 Mental Health, while it was positively correlated with education (see Table 2).

Weight was positively correlated with BMI, and it was negatively correlated with education, while it was not correlated with any psychological outcomes. Instead, BMI was only negatively correlated with education, GSI of SCL-90-R, and the BUT-A subscale of compulsive self-monitoring (see Table 2).

Marital status was only negatively correlated with education, work status was negatively correlated with the BUT-A subscale of depersonalization, and education was positively correlated with BUT-B PST (see Table 2).

BUT-A GSI and most of its subscales were positively correlated with SCL-90-R GSI, SCL-90-R PST, BDI II, and BHS and was negatively correlated with SF-12 scales (see Table 2). In addition, BUT-B PSDI, similarly to the BUT-B PST, was positively correlated with SCL-90-R GSI, SCL-90-R PST, and BDI II; however, it did not show significant correlation with BHS. Moreover, BUT-B PST was negatively correlated with SF-12 scales (see Table 2).

**Figure 1:**
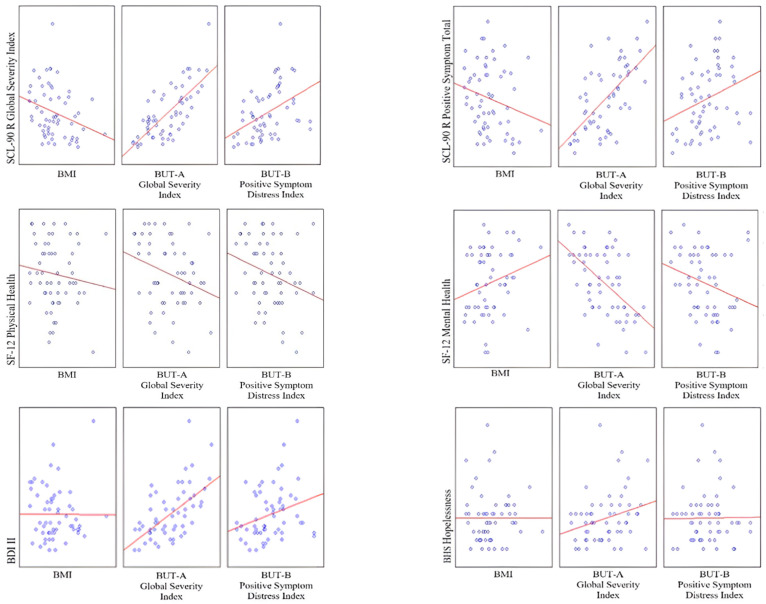
Graphical representation of associations between BMI, BUT-A GSI, and BUT-B PSDI with SCL-90 GSI, SCL-90 R PST, SF-12 Physical Health, SF-12 Mental Health, BDI II, and BHS.

The hierarchical regression analyses were performed to identify factors predicting psychological outcomes, i.e., SCL-90-R GSI, SCL-90-R PST, SF-12 Physic Health, SF-12 Mental Health, BDI II, and BHS. The independent variables were entered as follows: step 1 included BMI, while step 2 included BMI, BUT-A GSI, and BUT-B PSDI. The results of the regression analysis are depicted in the tables and in Figure 1.

The first hierarchical model was performed on SCL-90 GSI including two steps (first step: BMI as predictor; second step: BMI, BUT-A GSI, and BUT-B PSDI as predictors). In the first step, the significant model explained 6.7% of the variance, and the BMI resulted as a significant predictor. In the second step, the significant model explained 54.4% of the variance, and the BUT-A GSI resulted as a significant predictor (see Table 3 and Figure 1).

The second hierarchical model was performed on SCL-90-R PST including two steps (first step: BMI as predictor; second step: BMI, BUT-A GSI, and BUT-B PSDI as predictors). In the first step, the model resulted as not significant, and the BMI resulted as not a significant predictor. In the second step, the significant model explained 40.3% of the variance, and the BUT-A GSI resulted as a significant predictor (see Table 4 and Figure 1).

The third hierarchical model was performed on SF-12 Physic Health including two steps (first step: BMI as predictor; second step: BMI, BUT-A GSI, and BUT-B PSDI as predictors). In the first step, the model resulted as not significant, and the BMI resulted as not a significant predictor. Similarly, in the second step, the model was not significant, and BMI, BUT-A GSI, and BUT-B PSDI resulted as not a significant predictor (see Table 5 and Figure 1).

The fourth hierarchical model was performed on SF-12 Mental Health including three steps (first step: BMI as predictor; second step: BMI, BUT-A GSI, and BUT-B PSDI as predictors; third step: BMI, BUT-A GSI, BUT-B PSDI, and gender as predictors). In the first step, the model resulted as not significant, and the BMI resulted as not a significant predictor. In the second step, the significant model explained 29.5% of the variance, and the BUT-A GSI resulted as a significant predictor. In the third step, the model resulted as not significant, and the sociodemographic characteristic of gender resulted as not a significant predictor (see Table 6 and Figure 1).

Subsequently, the fifth hierarchical model was performed on BDI II including two steps (first step: BMI as predictor; second step: BMI, BUT-A GSI, and BUT-B PSDI as predictors). In the first step, the model resulted as not significant, and the BMI resulted as not a significant predictor. In the second step, the significant model explained 31.9% of the variance, and the BUT-A GSI resulted as a significant predictor (see Table 7 and Figure 1).

Finally, the last, sixth hierarchical model was performed on BHS including two steps (first step: BMI as predictor; second step: BMI, BUT-A GSI, and BUT-B PSDI as predictors). In the first step, the model resulted as not significant, and the BMI resulted as not a significant predictor. In the second step, the significant model explained 5.8% of the variance, and the BUT-A GSI resulted as a significant predictor (see Table 8 and Figure 1).

## 4. Discussion

In the present study, it was evaluated which aspect between the body mass index and the perception of one’s own body image may play an important role on psychophysical health in obese persons undergoing pre-bariatric surgery.

The main finding was that the body image, contrary to BMI, was a significant predictor of psychological health. Through the hierarchical regression, it was possible to highlight that the more people with obesity who are undergoing bariatric surgery that are satisfied with their own body image, the more likely they are to experience greater or better psychological well-being, in terms of less psychological distress, depression, and better mental health. This finding is consistent with the results of a previous study that investigated this type of positive, direct relationship between body image and psychological well-being [32,33]. Moreover, the hierarchical regressions showed that the BMI did not predict psychological well-being. In fact, the first hierarchical regression model showed the predictor role of BMI only with severity of the psychological distress complained by the individual, while, as soon as aspects related to the perception of one’s own body image are considered, it loses its predictive role toward the psychological distress.

Previous studies on obese patients showed a strong association between the BMI and the presence of psychological and social problems, and jointly a poor quality of life [34,35].

In fact, it has been reported that suffering from more severe obesity with a high BMI may influence the psychological well-being of people showing an increase in depression aspects and anxiety [35]. Unlike these results, the present study showed only a correlation between BMI and psychological distress. However, the results of present study seem to suggest that the effective real physical weight did not seem represent a risk factor per se of psychological distress. In support of these, a previous study investigating the same aspects presented in a sample of undergraduate students showed that BMI did not correlate with any psychosocial measures, unlike body image [6].

These results could be explained by the fact that, due to the multidimensional and complexity of body image, there may be several factors that influence the development of a negative body image, mainly in people suffering from obesity [36]. Beliefs and attitudes in considering our own body image, shape, and weight develop along life and are strongly influenced by social context [37]. In obese people, the perception of one’s own body image is not only dependent on a possible mismatch between perceived and desired body shapes, but also on the presence of mental images, emotions, and thoughts that lead to associated body appearance with personal value in the social context [17]. Indeed, these people are often made fun of for their weight and appearance, where the social stigma and discrimination can worsen their psychological well-being [38]. This may lead people with obesity to experience a real fear or phobia of fat and weight with a dissatisfaction with their body image.

Furthermore, in the present study, only the dissatisfaction for body image (BUT- A) was associated with psychological outcomes, while the hate for a specific part of one’s own body (BUT- B) showed lower grades of correlation with the psychological outcomes and, when inserted in the regression models, it did not show any significant effects. This result suggests that the preoccupation for specific parts of one’s own body seems to have a less relevant role compared to the global dissatisfaction for body image in the relationship with the psychological outcomes. A possible explanation of this result seems to be that the experience of one’s own weight is majorly related to the expectations that the individual and others have about body image more than to the objective weight with important clinical implications.

A further result that emerged from our study is related to the correlation between gender and mental health. At first, the association between these was significant and this would seem to confirm some previous studies where a worsening of psychological well-being was shown in women with obesity [39,40]. Subsequently, this effect would not appear to be confirmed by regression analyses and this could be motivated by the fact that the participants in the study experience, regardless of their gender, a condition of significant psychophysical distress due to their obesity to the extent that they perceive the choice of bariatric surgery as the only effective treatment option. Indeed, these results may contribute to a better understanding of the interaction between body image and obesity condition in the people undergoing bariatric surgery and could help clinical psychologists to create targeted interventions in a preoperative phase. In these patients, more considerations should be given to the experience of the perception of one’s own body image that cause the emotional suffering that afflicts so many of these individuals and consider it as a crucial variable in the outcome of interventions focused for the treatment of obesity.

The findings of this study should be interpreted with consideration due to some limitations. The first is that self-report measures were used. Furthermore, some participants might have falsified some of their answers to show a worse psychophysical state due to the assessment setting (psychological assessment for eligibility for bariatric surgery).

In addition, due to the reduced sample size, the study could be underpowered at detecting significant coefficients in the second stage of the regressions. Further studies should include a larger sample that would allow for complex statistical analyses, such as structural equation models. This would allow a better understanding of the role of body image in influencing the psychophysical health of patients during the preliminary phase of bariatric surgery.

More limitations were found in the lack of gender balance in the group of participants and in the recruitment of the sample in a single health center.

The present findings, which add to the existing literature on the perception of one’s body image in obese people, especially those who decide to undergo bariatric surgery, emphasize the need to investigate the role of body image especially in the long term when the body of these persons will undergo changes due to weight loss. The change in their body and the perception of this, if not addressed and processed correctly, could significantly affect their psychological health, risking a further deterioration in their general psychophysical health.

## 5. Conclusions

The body image dissatisfaction is more common in people with severe obesity, particularly those who choose bariatric surgery to cure their obesity. This study investigated the relative role of body image compared to body mass index in the association with psychophysical well-being in a clinical sample of patients undergoing bariatric surgery.

The results suggested a more relevant role of body image compared to the effective weight of BMI in the association with psychological and physical outcomes, suggesting the importance of considering body image in the assessment and treatment of obese patients requiring bariatric treatment.

## Figures and Tables

**Table 1:** Sociodemographic and psychological characteristics of participants (n = 59).

	Mean	SD	N(%) (*n* = 59)
Weight	115.62	22.89	
BMI baseline Class I (30.0–34.9) Class II (35.0–39.9) Class III (>40.0)	41.96	5.38	6(10.2%) 15(25.4%) 38(64.4%)
Education Primary School Secondary School University			35(59.3%) 17(28.9%) 7(11.8%)
Work Student Employed Unemployed Retired			4(6.9%) 28(47.3%) 26(44.1%) 1(1.7%)
Marital Status Single Married Separated Widowed			20(33.9%) 32(54.2%) 6(10.2%) 1(1.7%)
SCL-90-R SCL- 90-R GSI SCL-90-R PST	66.06 60.48	17.80 12.37	
BUT-A BUT-A GSI BUT-A Weight Phobia BUT-A Body Image Concern BUT-A Avoidance BUT-A Compulsive Self-monitoring BUT-A Depersonalisation	1.86 2.32 2.85 1.36 1.00 1.40	1.07 1.25 1.29 1.22 0.84 1.29	
BUT-B BUT-B PST BUT-PSDI	14.12 2.75	8.91 0.95	
SF-12 Total Physical Health Mental Health	33.20 13.93 19.27	6.63 3.37 4.34	
BDI II	10.59	7.72	
BHS	3.54	2.86	

**Table 2:** Correlational analyses (Pearson’s r) among the weight, BMI, sociodemographic characteristics, BUT, SCL-90-R, SF-12, BDI II, and BHS.

Variable	1	2	3	4	5	6	7	8	9	10	11	12	13	14	15	16	17	18	19	20	21
1. Gender	—																				
2. Weight	−0.75 ***	—																			
3. BMI	−0.56 ***	0.85 ***	—																		
4. Marital Status	−0.00	0.03	0.10	—																	
5. Work	0.19	−0.14	−0.10	−0.20	—																
6. Education	0.35 **	−0.34 **	−0.40 **	−0.40 ***	0.09	—															
7. BUT-A GSI	0.12	−0.11	−0.16	0.17	−0.17	0.08	—														
8. BUT-A Weight Phobia	0.09	−0.16	−0.19	0.13	−0.08	0.00	0.91 ***	—													
9. BUT-A Body Image Concern	0.08	−0.01	−0.09	0.17	−0.12	0.07	0.92 ***	0.84 ***	—												
10. BUT-A Avoidance	0.08	−0.02	−0.06	0.05	−0.18	0.09	0.84 ***	0.65 ***	0.71 ***	—											
11. BUT-A Compulsive Self-monitoring	0.07	−0.23	−0.28 *	0.14	−0.13	0.15	0.73 ***	0.68 ***	0.57 ***	0.49 ***	—										
12. BUT-A Depersonalisation	0.20	−0.14	−0.15	0.23	−0.30 *	0.07	0.83 ***	0.63 ***	0.67 ***	0.76 ***	0.56 ***	—									
13. BUT-B PST	0.16	−0.11	−0.16	−0.15	−0.05	0.26 *	0.54 ***	0.42 ***	0.45 ***	0.53 ***	0.48 ***	0.51 ***	—								
14. BUT-B PSDI	0.05	−0.01	0.04	0.18	−0.08	−0.07	0.56 ***	0.53 ***	0.57 ***	0.41 ***	0.39 **	0.41 ***	0.203	—							
15. SCL−90-R GSI	0.18	−0.19	−0.29 *	0.07	−0.15	0.06	0.73 ***	0.63 ***	0.59 ***	0.73 ***	0.53 ***	0.70 ***	0.611 ***	0.42 ***	—						
16. SCL−90R PST	0.15	−0.08	−0.21	0.10	−0.20	0.00	0.65 ***	0.56 ***	0.52 ***	0.65 ***	0.42 ***	0.64 ***	0.565 ***	0.29 *	0.92 ***	—					
17. SF−12 Physical Health	−0.10	−0.00	−0.12	−0.14	−0.03	0.13	−0.29 *	−0.23	−0.25	−0.31 *	−0.26 *	−0.20	−0.176	−0.27 *	−0.39 **	−0.44 ***	—				
18. SF−12 Mental Health	−0.27 *	0.22	0.23	−0.03	−0.01	−0.10	−0.56 ***	−0.48 ***	−0.45 ***	−0.63 ***	−0.34 **	−0.48 ***	−0.548 ***	−0.26 *	−0.69 ***	−0.65 ***	0.47 ***	—			
19. SF−12 Total Score	−0.23	0.14	0.09	−0.09	−0.02	−0.00	−0.51 ***	−0.43 ***	−0.42 ***	−0.57 ***	−0.35 **	−0.42 ***	−0.448 ***	−0.30 *	−0.65 ***	−0.65 ***	0.82 ***	0.89 ***	—		
20. BDI II	0.16	0.01	−0.00	0.24	−0.14	−0.09	0.58 ***	0.46 ***	0.49 ***	0.61 ***	0.41 ***	0.57 ***	0.398 **	0.28 *	0.67 ***	0.65 ***	−0.49 ***	−0.64 ***	−0.67 ***	—	
21. BHS	−0.09	0.05	0.00	0.02	−0.04	−0.14	0.27 *	0.23	0.22	0.39 **	0.07	0.23	−0.020	0.01	0.38 **	0.39 **	−0.20	−0.38 **	−0.35 **	0.38 **	—

Note. BMI = Body Mass Index; SCL-90-R GSI = Symptom Checklist-90-Revised Global Severity Index; SCL-90 R PST = Positive Symptom Total; BUT = Body Uneasiness Test; SF-12 = 12-item Short Form Survey; BDI II = Beck Inventory Scale II; BHS = Beck Hopelessness Scale. * = *p* < 0.050; ** = *p* < 0.010; *** = *p* < 0.001.

**Table 3:** First hierarchical regression models including two steps, with BMI as predictor in first step; BMI, BUT-A GSI, and BUT-B PSDI as predictors of SCL-90 GSI in second step.

Outcome: SCL-90 R GSI									
			Step 1				Step 2		
			B	β	*p*		B	β	*p*
Independent Variables	BMI		−0.030	−0.288	0.027		−0.018	−0.180	0.053
	BUT-A GSI						0.361	0.673	<0.001
	BUT-B PSDI						0.031	0.054	0.623
									
Model	R	0.288				0.754			
Summary	R^2^	0.083				0.568			
	Adjusted R^2^	0.067				0.544			
	F	5.15				24.08			
	Sig. of the model	0.027				<0.001			
	F^2^	0.090				1.314			
	R^2^ change					0.485			

**Table 4:** Second hierarchical regression models including two steps, with BMI as predictor in first step; BMI, BUT-A GSI, and BUT-B PSDI as predictors of SCL-90 PST in second step.

Outcome: SCL-90 R PST									
			Step 1				Step 2		
			B	β	*p*		B	β	*p*
Independent Variables	BMI		−0.766	−0.215	0.101		−0.363	−0.102	0.332
	BUT-A GSI						12.551	0.675	<0.001
	BUT-B PSDI						−1.667	−0.083	0.508
									
Model	R	0.215				0.659			
Summary	R^2^	0.046				0.434			
	Adjusted R^2^	0.030				0.403			
	F	2.77				14.05			
	Sig. of the model	0.101				<0.001			
	F^2^	0.048				0.767			
	R^2^ change					0.387			

**Table 5:** Third hierarchical regression models including two steps, with BMI as predictor in first step; BMI, BUT-A GSI, and BUT-B PSDI as predictors of SF-12 Physical Health in second step.

Outcome: SF-12 Physical Health									
			Step 1				Step 2		
			B	β	*p*		B	β	*p*
Independent Variables	BMI		−0.076	−0.122	0.358		−0.097	−0.156	0.236
	BUT-A GSI						−0.786	−0.240	0.131
	BUT-B PSDI						−0.456	−0.128	0.410
									
Model	R	0.122				0.349			
Summary	R^2^	0.015				0.122			
	Adjusted R^2^	−0.002				0.074			
	F	0.858				2.551			
	Sig. of the model	0.358				0.065			
	F^2^	0.015				0.139			
	R^2^ change					0.107			

**Table 6:** Fourth hierarchical regression models including three steps, with BMI as predictor in first step; BMI, BUT-A GSI, and BUT-B PSDI as predictors in second step; BMI, BUT-A GSI, BUT-B PSDI, and gender as predictors of SF-12 Mental Health in third step.

Outcome: SF-12 Mental Health													
			Step 1				Step 2				Step 3		
			B	β	*p*		B	β	*p*		B	β	*p*
Independent Variables	BMI		0.186	0.231	0.078		0.110	0.137	0.231		0.021	0.027	0.841
	BUT-A GSI						−2.373	−0.563	<0.001		−2.394	−0.568	<0.001
	BUT-B PSDI						0.240	0.052	0.699		0.318	0.069	0.606
													
	GENDER										−1.908	−0.193	0.151
												
Model Summary	R	0.231				0.576				0.597			
	R^2^	0.053				0.331				0.357			
	Adjusted R^2^	0.036				0.295				0.309			
	F	3.21				9.09				7.49			
	Sig. of the model	0.078				<0.001				<0.001			
	F^2^	0.056				0.495				0.555			
	R^2^ change					0.278				0.025			

**Table 7:** Fifth hierarchical regression models including two steps, with BMI as predictor in first step; BMI, BUT-A GSI, and BUT-B PSDI as predictors of BDI II in second step.

Outcome: BDI II									
			Step 1				Step 2		
			B	β	*p*		B	β	*p*
Independent Variables	BMI		−0.007	−0.005	0.972		0.151	0.105	0.347
	BUT-A GSI						4.895	0.653	<0.001
	BUT-B PSDI						−0.761	−0.093	0.483
									
Model	R	0.005				0.595			
Summary	R^2^	0.020				0.354			
	Adjusted R^2^	−0.017				0.319			
	F	0.001				10.068			
	Sig. of the model	0.972				<0.001			
	F^2^	0.020				0.548			
	R^2^ change					0.354			

**Table 8:** Sixth hierarchical regression models including two steps, with BMI as predictor in first step; BMI, BUT-A GSI, and BUT-B PSDI as predictors of BDI II in second step.

Outcome: BHS									
			Step 1				Step 2		
			B	β	*p*		B	β	*p*
Independent Variables	BMI		0.001	0.002	0.989		0.041	0.077	0.561
	BUT-A GSI						1.123	0.404	0.013
	BUT-B PSDI						−0.651	−0.216	0.172
									
Model Summary	R	0.002				0.327			
	R^2^	0.000				0.107			
	Adjusted R^2^	−0.017				0.058			
	F	0.059				2.19			
	Sig. of the model	0.989				0.099			
	F^2^	0.000				0.120			
	R^2^ change					0.107			

## Data Availability

All subjects gave their informed consent for inclusion before they participated in the study.
